# Editorial and Miscellaneous

**Published:** 1864-06

**Authors:** 


### EDITORIAL AND MISCELLANEOUS.

[The following interesting article was received too late to be placed among original communications.—Eds.]

*Creosote and Arsenic in the Treatment of Skin Diseases.*—From the well-known prevalence and obstinate character of some of the cutaneous diseases of the North-Western States, frequently resisting alike the efforts of well-directed medical skill, the never-failing unguents, lotions, and alterative secretion regulating secret remedies of the presumptuous charlatan, and much-lauded specific nostrums of the patent medicine vendor, I desire, through the medium of your widely-circulating journal, to commend to the consideration of the profession, after an experience of fifteen years, the above articles in the treatment of cutaneous diseases.

Having kept no record of cases I have no data but memory from which to write. I shall, therefore, make a brief general statement of the manner in which I have been in the habit of using the above articles, and if the statements which I may make should prove of benefit to any one of the profession, or any of the afflicted, my end will have been attained.

I have, during the time specified, treated many cases of these diseases, of various grades, and have found more good resulting from the use of creosote than from all the other articles which I have used. The diseases in which I have found it more particularly useful, are impetigo and porrigo, and more especially of these, porrigo larvalis of Millan and Bateman, or impetigo larvalis of the French writers; this variety affecting young children during the period of dentition, is a source of much annoyance both to nurses and doctors, to say nothing of the more serious consequences which may occasionally result from its long continuance, where it seems to baffle all the skill of practitioner and nurse.

This variety (for a description see Wood's Prac. of Med.,) sometimes discharges fluid so copiously that scabs can not form; it is in such cases that I have found the creosote to act most advantageously, and also in the particular cases of eruption of the scalp. I have been much gratified with its results, indeed, it has given entire satisfaction in almost every case where I have been able to fairly test its virtues. The manner in which I have found it to act most beneficially is in form of an ointment, made according to the following formula:—

℞. Adeps preparata, ℥j; Creosote, gtt. xxv. Mix on a clean tile or plate until the two are thoroughly incorporated. Apply the ointment with a camel's hair pencil or the tip of the finger to the parts affected, twice a day, cleansing it well with fine soap and rain water once a day. If the ointment in this proportion should be too strong for the particular case to which it is applied, the inflammation will be increased; this should be an indication for a lessening of the creosote; if on the contrary it is too weak, the eruption will heal very slowly or not at all, which is an indication for greater strength. The constitutional symptoms, if any are present, and it frequently so occurs, should be met at the same time with appropriate remedies, and it is here that I have found arsenic, in the form of Fowler's Solution, more valuable than all the other alteratives and entrophics which I have used.

This may be very safely administered to small children if the nurses are sufficiently warned of the care necessary to be taken, to give only the prescribed dose. The manner in which I have usually given it is as follows: To a child from 2 to 6 months old, gtt. j; from 6 to 18 months, gtt. iss; from 18 to 24 months, gtt. ij; two or three times in 24 hours, increasing with the age. I have been in the habit of continuing the use of the solution for a length of time varying from two to six months, which practice would seem to suggest itself as being beneficial on account of the well-known properties of arsenic in promoting nutrition. J. ELLES LYONS, M. D.

HUNTINGTON, Ind.

---

At a recent meeting of the Chicago Medical Society, Dr. Bartlett presented a means of using chloroform, when its application must of necessity be frequent or immediate, as in convulsions, hooping cough, neuralgia, labor, etc. He recommended it also as a matter of economy. By its use the chamber of the patient is kept comparatively free from the odor of chloroform, to many disagreeable or sickening.

Into a four-ounce gallipot, Dr. B. fits a cup-shaped sponge, retaining it in place by a transverse stay of wood. The anæsthetic being poured upon the sponge, the pot is placed inverted in a saucer containing a little water (or mercury). The tention of chloroform vapor not being great and it being sparingly soluble in water, but little is lost. The sponge may be successfully used hours after the pouring on of the anæsthetic.

---

*Illinois State Homœopatic Association.*—This city has recently been favored with a meeting of this organization, and of its transactions we find an elaborate report in the *Chicago Tribune.* From this, we learn that one of the fraternity, having treated a case a long time without any benefit to the patient, and it seems without knowing what the disease was, hit upon the happy expedient of taking him before the assembled wisdom of the Association to find out, whereupon the report says: "Dr. Ludlam moved that the physicians ballot for a diagnosis. The result of the vote was as follows: psoriasis inveterata, 5; skin disease, 1; eczema rubra, 5; tetter, 1; rara avis in terra, 1; leprosy, 3; natrum muriaticum, 1; psora, 1; blank, 3; Persian leprosy, 2."

Could this question be determined by the weight, rather than the number, of opinions, we have no doubt the *savant* who diagnosed "skin disease," would have the question by an overwhelming preponderance. The President, Dr. Smith, said he had cured a case of phthisis pulmonalis, by administering a single dose of phosphorus. "The patient was the daughter of a homœopathic physician, and as there were no allopathic prejudices to be overcome, this might account for the rapidity of the cure." The points of interest omitted in the report of this case are,—the number and magnitude of the cavities that were present in the lungs, and what influence "allopathic prejudices" have on the action of phosphorus.

This nearly equals a case related by Hahnemann, in which powerful effects were produced on a patient, by smelling at a vial, in which a small pill, moistened with a medicine of the thirtieth attenuation, had been placed twenty years before.

Dr. Beebe introduced a resolution that is entirely inexplicable to us, which goes on to recite, "That while we revere and respect the chief Magistrate of the nation, and will ever prove loyal to the government, we deprecate and earnestly disapprove his compromise of character and self-respect in the utterance of an anecdote accredited to him." This resolution leads us to suspect that this meeting was not entirely a scientific one, but that under this cloak were concealed political objects, designed to overthrow the hopes of Mr. Lincoln for another term. It was clearly unjust to the President to censure him for an "anecdote accredited to him," without specifying what anecdote it was, and thereby give him an opportunity either to deny it or justify himself to the parties who feel themselves so much aggrieved. Why did they not put the "anecdote" in the "belly of the resolution," as Mr. Benton would have said. We have heard but one story accredited to Mr. Lincoln, relating to homœopathists, and what that one can have to do with the "loyalty" of the members of the Association, we can not conceive. We write in a state of painful doubt and curiosity as to what the anecdote was.

---

*Medical Graduates for* 1864.—Jefferson Medical College, 124; University of Pennsylvania, 101; Bellevue Hospital Medical College, 95; Rush Medical College, Chicago, 80; College of Physicians and Surgeons, N. Y., 72; Medical Department of University, N. Y., 59; Buffalo Medical College, 41; Starling Medical College, 32; Medical College of Ohio, 31. Cincinnati College of Medicine and Surgery, 30; Chicago Medical College, 17; Med. Department of University of Pacific, 7.

---

Some one has said, "Try all things; hold fast that which is good."—*Ohio Med. and Surg. Journal.*

To attribute one of the most familiar injunctions of Paul to "*some one*," reminds us of a preacher we used to hear in the days of the early settlement of Illinois, who in one of his sermons was urging the importance of giving to the young, proper instruction, and feeling in his own person the inconvenience growing out of a neglect of this duty, and wishing to enforce his views by the sanction of Scripture, did it thus:—"For, my friends, it is written in this inspired Book," placing his hand at the same time, with great emphasis, on the Bible, "Just as the twig is bent the tree is inclined."

---

*Mortality Among Hospital Physicians.*—Five members of the resident medical staff of Bellevue Hospital, city of New York, have paid the forfeit of their lives, and ten more have barely escaped death, since the 1st of May last, from fever, contracted in the discharge of their duties to the patients in that institution. The *Medical Times* charges the mischief to imperfect ventilation.

